# Electrochemical Detection of Melatonin in the Presence of Dopamine on a Ce-Doped Poly(bromocresol purple)-Modified Electrode

**DOI:** 10.1021/acsomega.5c04226

**Published:** 2025-10-09

**Authors:** Hülya Öztürk Doğan, Neslihan Çelebi, Arzu Kavaz Yüksel

**Affiliations:** † Department of Chemistry and Chemical Processing Technologies, Erzurum Vocational College Erzurum, Atatürk University, Erzurum 25240, Turkey; ‡ Department of Nanoscience and Nanoengineering, 37503 Ataturk University, Erzurum 25240, Turkey; § Vocational School of Technical Sciences, Department of Food Technology, Ataturk University, Erzurum 25240, Turkey

## Abstract

In this study, bromocresol purple was electropolymerized on the surface of a pencil graphite electrode (PGE) and electrochemically doped with cerium (Ce) nanoparticles. The Ce-doped poly­(bromocresol purple) (Ce/PBCP)-modified PGE was characterized using energy dispersive X-ray spectroscopy, scanning electron microscopy, and X-ray photoelectron spectroscopy. The prepared Ce/PBCP electrode was investigated for the electrochemical determination of melatonin in the presence of dopamine. The electrochemical activity of the Ce/PBCP electrode was compared with that of undoped PBCP, Ce/PGE, and bare PGE. The peak potential and peak current for melatonin oxidation were +800 mV and 820 μA cm^–2^, respectively. The detection limit of melatonin was 0.038 μM, and the Ce/PBCP electrode exhibited high activity in the presence of interfering species. In addition, the use of a Ce/PBCP electrode for detecting melatonin in a terebinth sample, a real food sample, was also investigated, and the produced electrode demonstrated high performance.

## Graphical abstract

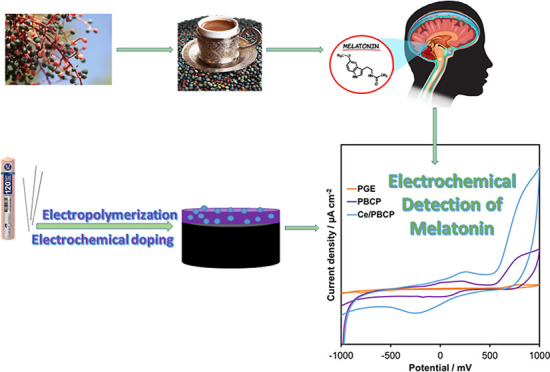

## Introduction

Melatonin is the specific name of the molecule N-acetyl-5-methoxy tryptamine.^1,2^ Melatonin, a derivative of serotonin, plays a crucial role in the circadian rhythm of living organisms and is the active ingredient in drugs used to treat sleep disorders.^3–6^ Additionally, melatonin is an antioxidant agent with anti-inflammatory properties, playing a crucial role in the immune system.^7,8^ Its synthesis occurs in not only mammals but also in single-celled organisms, fungi, and plants.^9^ Moreover, some coffee, tea, and herbal teas also contain melatonin.^10,11^ Various analytical techniques, including electrophoresis, mass spectrometry, and electrochemical detection, have been employed for the determination of melatonin in pharmaceutical formulations and biological fluids.^12–16^ Among these techniques, electrochemical determination is an advantageous method with its low detection limit, wide linear range, and low device cost, as well as being simple, applicable, rapid, and selective.^17–19^ However, modification or doping of the electrode surface to enhance electrochemical response may provide significant advantages.^20–22^

Conductive polymer surfaces have been widely used as substrates in electrochemical sensing, providing a more conducive and larger surface area.^23^ Bromocresol purple (BCP; 5′,5″-dibromo-*o*-cresolsulfonephthalein), used as a pH indicator, is a water-soluble crystalline dye.^24^ BCP can be easily polymerized electrochemically and forms a poly­(bromocresol purple) (PBCP) film on the electrode surface.^25–27^ The presence of PBCP on the electrode surface offers advantages, including increased conjugated bond density, a high surface area, a higher number of active sites, and improved conductivity.^28^ These properties have led to the use of PBCP-modified electrodes in the electrochemical determination of purine derivatives and hydrazine.^21^ Additionally, several studies in the literature have utilized PBCP-modified electrodes for the electrochemical determination of various analytes.^26,29–31^ However, PBCP/PGE, obtained by modifying pencil graphite electrodes (PGE) with PBCP, were used for the first time for the electrochemical detection of melatonin.

Cerium (Ce), the most abundant rare-earth metal in the Earth’s crust, has been preferred in sensor applications due to its biocompatibility, as well as its chemical and thermal stability.^32,33^ Moreover, the switchable redox reactivity, high catalytic activity, high sensitivity, fast response time, and high stability of nanosized cerium have made it advantageous for use in electrochemical sensors.^34–37^ To the best of our knowledge, no study has been published reporting the simultaneous electrochemical determination of dopamine and melatonin using PBCP-modified PGE doped with Ce nanoparticles. In the present work, we describe the electrochemical synthesis of Ce-doped PBCP-modified electrodes (Ce/PBCP) and investigate the performance of this modified surface for the electrochemical detection of melatonin for the first time. We also evaluate its analytical performance for the amperometric quantitative determination of melatonin.

## Experimental Section

### Chemicals and Apparatus

Cerium­(IV) sulfate (Ce­(SO_4_)_2_), bromocresol purple, sodium phosphate dibasic acid (Na_2_HPO_4_), dopamine hydrochloride (DA), and melatonin were purchased from Sigma-Aldrich (U.S.A) and used directly. All solutions were prepared using ultrapure water. For melatonin detection, a 0.1 M Na_2_HPO_4_ solution with a pH of 7 was selected as the phosphate buffer solution. A Gamry Interface 1010 model potentiostat/galvanostat and a three-electrode cell system were used in all electrochemical experiments. In electrochemical measurements, the working electrode, the counter electrode, and the reference electrode were a pencil graphite electrode (PGE, Faber Castell, 0.7 mm), a Pt wire, and Ag/AgCl, respectively. The oxidation potential of melatonin was determined using cyclic voltammetry. Cyclic voltammograms (CVs) were recorded in the potential range between −1 V and +1 V. Amperometric determination was examined at different melatonin concentrations. Electrochemical impedance spectroscopy (EIS) was performed at +0.2 V constant potential and an AC voltage amplitude of 10 mV in the frequency range from 0.1 Hz to 100 kHz.

Binding energies of the modified electrodes were investigated by using X-ray photoelectron spectroscopy (XPS, Specs Flex XPS model) with an Al Kα excitation source. Morphological characterization was performed by field emission scanning electron microscopy (FESEM) equipped with a ZEISS Gemini Sigma 300 model and energy dispersive X-ray spectroscopy (EDS).

### Preparation of Modified Electrodes

The PGE selected as the working electrode was cleaned by sonication in an ethanol medium. First, a PBCP film was prepared on the PGE surface by electropolymerization, and then electrochemical doping with Ce nanoparticles was performed. Electropolymerization of BCP on the PGE surface was carried out according to a procedure reported in the literature.^31,38,39^ For this purpose, a 5 mM BCP monomer solution in 0.1 M phosphate buffer (pH 6) was prepared. A PBCP film was synthesized on the PGE surface using a cyclic voltammetry technique from −600 to +1800 mV for 10, 20, and 30 potential cycles. In the CV recorded for the electropolymerization of BCP (CV in the inset of Scheme 1), the oxidation peak was observed at +0.5 V. The peak current of the monomer decreased with increasing number of cycles. Previous reports supported this electrochemical behavior and the formation of the PBCP film.^39^ Ce doping was carried out at −800 mV using a PBCP-modified PGE as the working electrode at different deposition times in a 5 mM Ce­(SO_4_)_2_ electrolyte. Similar electrochemical behavior for Ce deposition was also obtained on the carbon nanotube-based composite-modified glassy carbon electrode surface.^40^ It has been reported that Ce can be deposited at potentials more negative than −0.7 V. Scheme 1 illustrates the preparation procedure for Ce/PBCP-modified electrodes.

**Scheme 1:**
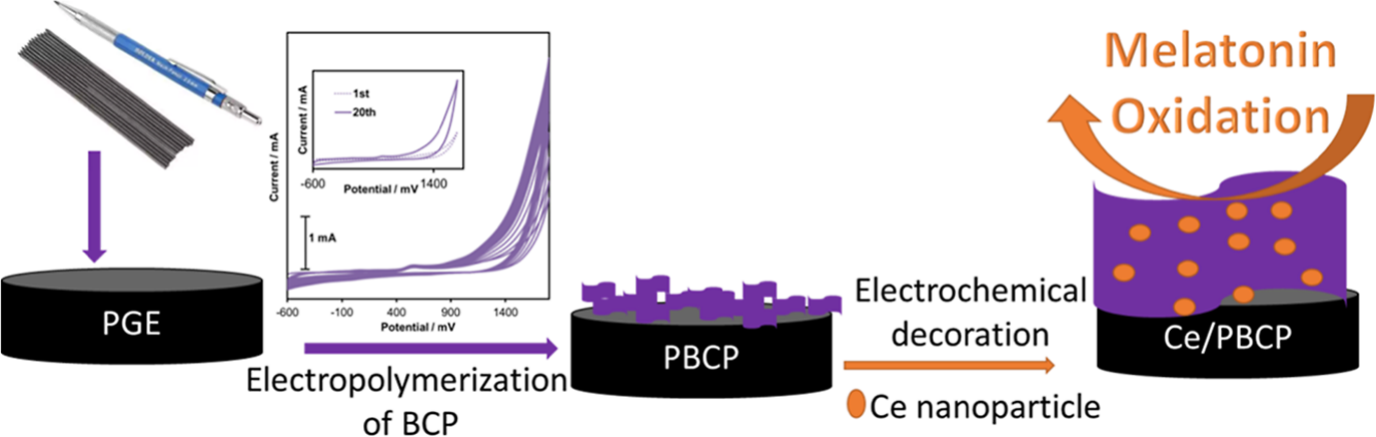
Preparation Procedure of Ce/PBCP-Modified Electrodes

### Real Sample Analysis

Turpentine coffee was selected as a real food sample. Two grams of turpentine coffee, obtained from the old bazaar in Mardin, Turkey, was dissolved in 12.5 mL of 0.1 M phosphate buffer (pH 7) and filtered to remove residues. The measurements were recorded with the collected supernatant. Spectroscopic analysis was performed using a Beckman Coulter DU730 Life Science model UV–vis spectrometer for method comparison.

## Results and Discussion

### Characterization of the Ce/PBCP Electrode

The XPS spectrum was recorded for the structural analysis of Ce-doped PBCP. High-resolution XPS spectra of Ce 3d, C 1s, O 1s, Br 3d, and S 2p core levels, originating from PBCP and Ce present in the Ce/PBCP electrode, are shown in Figure 1. In the core-level XPS spectrum of Ce (Figure 1a), the 3d spectrum peak is very useful for distinguishing metallic Ce or CeO_2_. In previous reports, the metallic Ce peak was observed at 883.7 eV, while the CeO_2_ peak varied in the region of 881.8–882.4 eV.^41^ The energy separation of the Ce 3d3/2 and Ce 3d5/2 peaks was observed to be ∼18.5 eV, supporting the formation of a metallic structure. Furthermore, the absence of satellite peaks confirmed that Ce doping was not in the form of CeO_2_. XPS analysis can also be used to quantitatively determine the elements. The XPS results indicated that the weight percentages of C, O, Br, S, and Ce were 54.1%, 40.4%, 2.3%, 1.1%, and 2.1%, respectively. Core-level XPS spectra for C, O, Br, and S were deconvoluted by using the Gaussian fit program. The high percentage of C is due to the use of PGE as a substrate. Five types of carbon bonds originating from different C bonds were detected in the C 1s spectrum of Ce/PBCP (Figure 1b). As shown in Figure 1b, in the C 1s spectrum, in addition to C=C (284.2 eV) and C–C (285.3 eV) bonds, peaks in the region of 286.0–288.8 eV were observed for C–O, C–Br, and C–S bonds. Previous reports also supported the existence of the BCP structure for C–Br and C–S bonds.^42^ The O 1s spectrum of Ce/PBCP (Figure 1c) was deconvoluted to contain two main peaks. The O 1s component peaks at binding energies of 532.1 and 530.6 eV were assigned to S=O and S–O bonds, respectively. The high-resolution spectrum recorded for the Br 3d core level (Figure 1d) contains peaks at 70.1 and 67.7 eV, assigned to C–Br 3d3/2 and C–Br 3d5/2, respectively. Moreover, the S 2s spectrum contains two peaks corresponding to the C–S–O (167.0 eV) and C–S=O (168.6 eV) bonds (Figure 1e). As expected, the presence of S 2p and Br 3d peaks in the XPS spectrum of the Ce/PBCP electrode confirmed the presence of PBCP on the PGE surface (Figure 1d–e).

**Figure 1:**
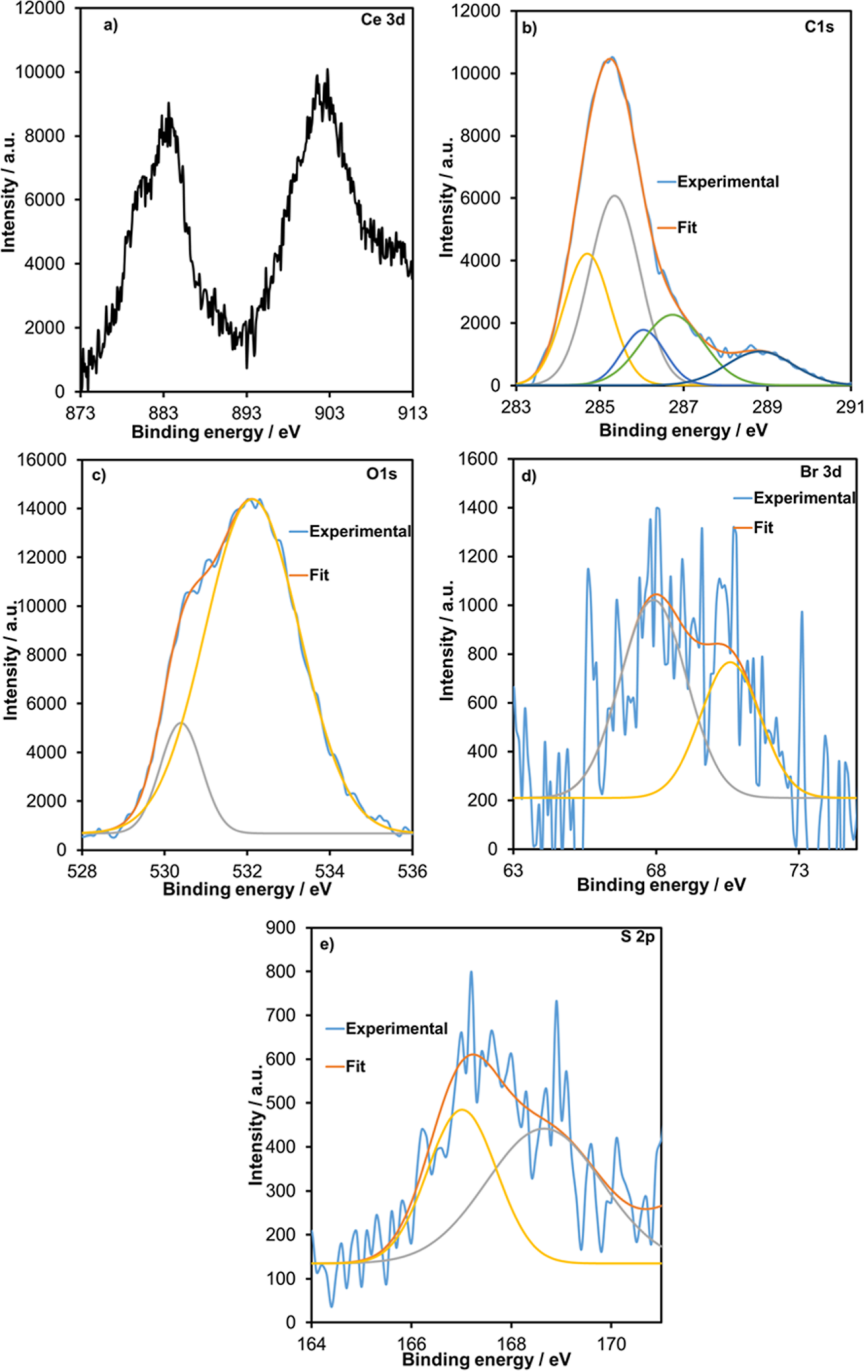
XPS spectra of Ce/PBCP electrodes. Core-level XPS for Ce 3d (a), C 1s (b), O 1s (c), Br 3d (d), and S 2p (e) of Ce/PBCP.

Ce/PBCP electrodes exhibiting the highest electrocatalytic activity for melatonin sensing were characterized. In order to characterize the morphological changes that occur on the surface of the PBCP electrode with Ce doping, FESEM images were recorded before (Figure 2a) and after (Figure 2b) Ce doping. Unmodified PGE was previously reported to have flake-like structures of graphite. At 5000× magnification in the FESEM images, it was observed that PBCP was deposited as a film on the PGE surface. After Ce doping, deposits in the form of small spherical particles were detected on the PBCP surface.

**Figure 2:**
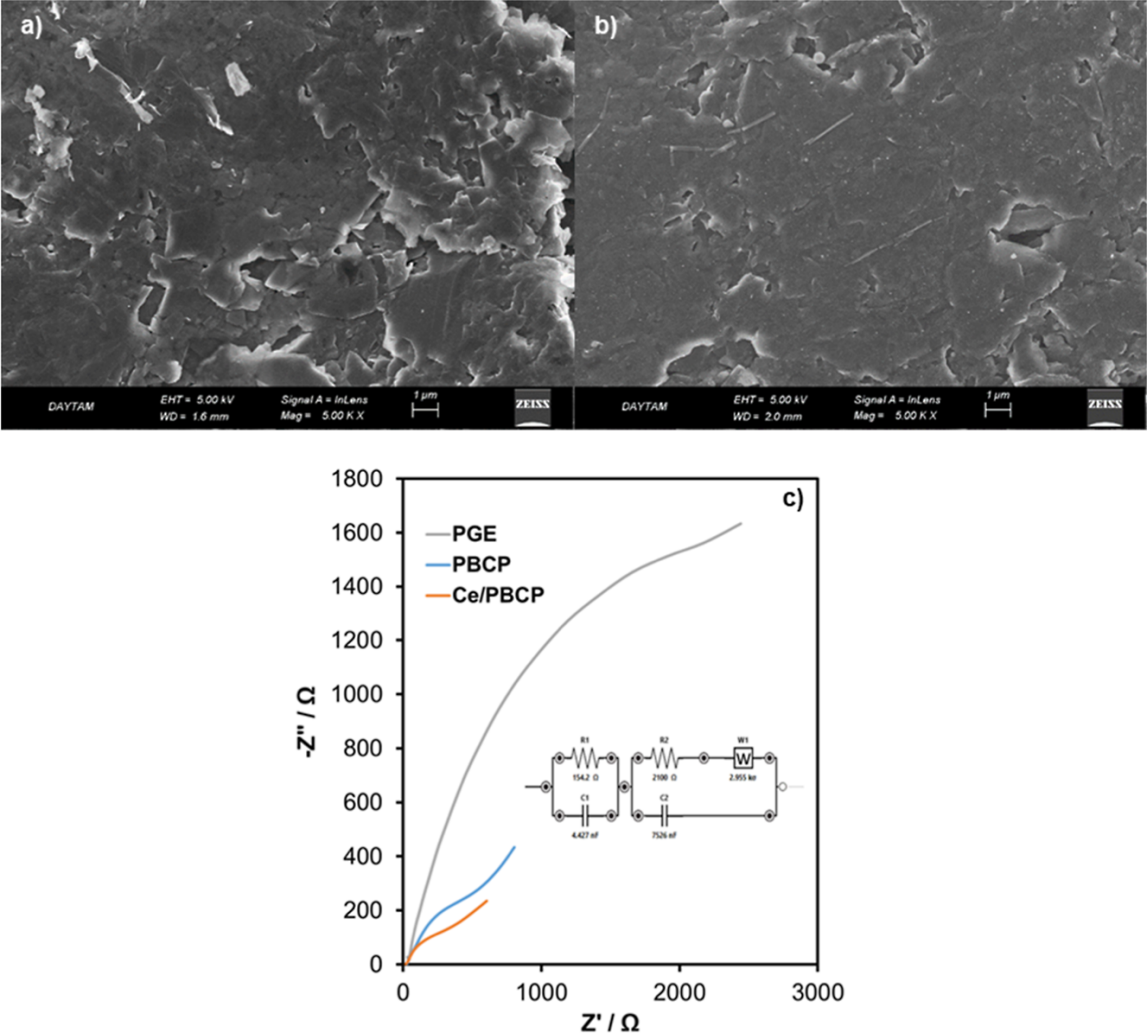
FESEM images of PCBP (a) and Ce/PBCP (b) electrodes. EIS spectra of PCBP and Ce/PBCP electrodes (c).

The electrochemical characterization of the produced Ce/PBCP and PBCP electrodes was investigated by EIS with a related equivalent circuit (Figure 2c). In the EIS spectra recorded in a 0.1 M KCl electrolyte containing a 1 mM ferri/ferro redox couple, the solution resistance was determined as 20.1 Ω. In EIS equivalent circuit modeling, the electrode charge transfer resistance (R) and the electrical double layer capacitance (C) are used to represent the electrode overpotential. They are traditionally assimilated with parallel resistors and capacitor element. The Warburg element (W), associated with diffusion or mass transfer resistance, must also be considered in the polarization impedance of carbon-based and porous electrodes such as PGE. The charge transfer resistances of the produced electrodes were determined using the semicircle diameter in the Nyquist curve. Accordingly, the charge transfer resistances for Ce/PBCP, PBCP, and PGE were calculated as 287 Ω, 595 Ω, and 2200 Ω, respectively. The smaller charge transfer resistance of Ce/PBCP in the EIS spectra indicated that it could facilitate reasonable charge transfer at the electrode/electrolyte interface.

### Electrochemical Detection of Melatonin on the Ce/PBCP Electrode

For the electrochemical detection of melatonin on the Ce/PBCP electrode surface, a 0.1 M phosphate buffer electrolyte with a pH of 7, containing 1 mM melatonin, was used. The oxidation potential of melatonin was investigated using CV. For this purpose, the CV graph was recorded from −1 V to +1 V at a scanning speed of 100 mV/s. First, the effect of the cycle number of PBCP on the melatonin oxidation peak was studied (Figure 3a). The oxidation currents of the PBCP electrodes produced after 10, 20, and 30 cycles were compared. The current values for 10, 20, and 30 cycles were determined as 226, 286, and 185 μA cm^–2^, respectively. The highest current response was obtained in the PBCP prepared in 20 cycles. Current studies using PBCP as an electrode material in biosensor applications have confirmed that the 20-cycle polymer exhibits the highest electrocatalytic activity.^31,38,39^ Afterward, Ce doping was carried out at different times on the PBCP electrode surface, which was synthesized through 20 cycles. The melatonin responses of PBCP electrodes doped with Ce for 30 s, 1 min, and 3 min are shown in Figure 3b. The melatonin oxidation currents for 30 s, 1 min, and 3 min Ce-doping times were 708, 820, and 628 μA cm^–2^, respectively.

**Figure 3:**
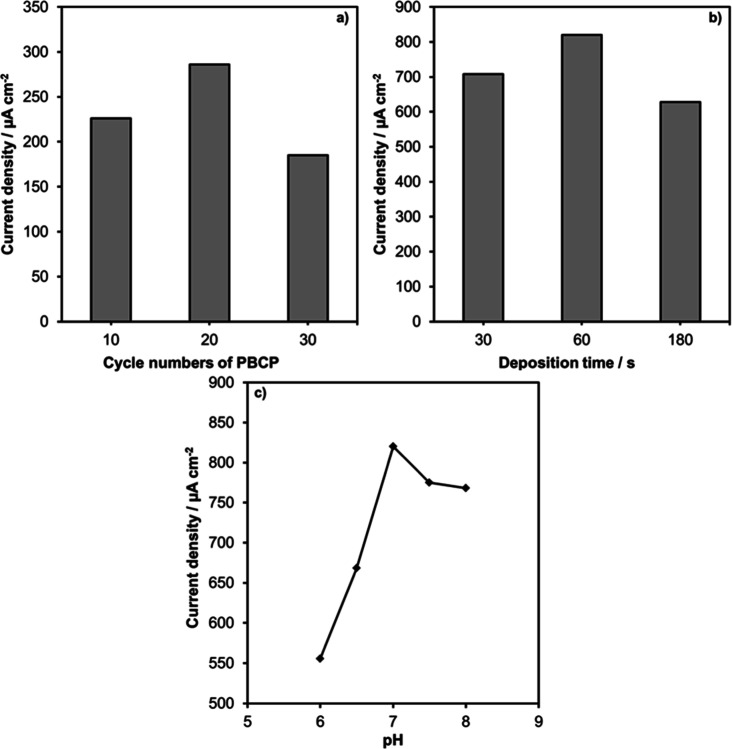
Effect of PBCP cycle number (a), Ce deposition time (b), and pH (c) on melatonin oxidation current.

The electrochemical behavior of melatonin generally depends on the pH of the electrolyte.^43^ Therefore, the CV plots of melatonin at the Ce/PBCP electrode in phosphate buffer solution were recorded in the pH range from 6 to 8. The maximum current densities of melatonin oxidation in CV were plotted versus pH (Figure 3c). As can be seen, the oxidation current increased with increasing pH and reached a maximum at pH 7. At pH values greater than 7, the current decreased significantly, indicating that 7 was the ideal pH value for the supporting electrolyte.

To compare the positive properties of Ce doping on the PBCP electrode, the melatonin responses of PGE, PBCP, and Ce/PBCP electrodes were overlapped in the same graph (Figure 4a). The Ce/PBCP electrode exhibited approximately 3.2 and 17.8 times higher current density as melatonin oxidation current compared to undoped PBCP and bare PGE, respectively. Furthermore, CV plots were recorded at scan rates of 5, 10, 25, 50, 100, 250, and 500 mV/s to investigate the dependence of current density on the scan rate (Figure 4b). The square root of scan rate vs current density (*v* ^1/2^–*j*) and scan rate vs current density (*v*–*j*) plots were created using the CVs shown in Figure 4b. Although the change of *j* with *v* ^1/2^ appears linear, the *R* ^2^ value of the *v*–*j* plot is higher. Therefore, the *v*–*j* change was evaluated. However, it indicated that adsorption of melatonin could be possible on the Ce/PBCP electrode surface. This is remarkably consistent with previous reports, suggesting that diffusion-controlled electrooxidation is present on carbon-based electrodes with adsorption properties.^44,45^ An increase in the current density was obtained with the increase in the scan rate, indicating that the oxidation of melatonin occurred through a surface-controlled process. The surface-controlled oxidation process of melatonin is compatible with most electrodes reported in the literature.^45,46^ The increase in current was not due to the increase in the number of active centers on the electrode surface resulting from Ce doping, demonstrating that the nanostructure had a synergistic effect on melatonin sensing.^34^

**Figure 4:**
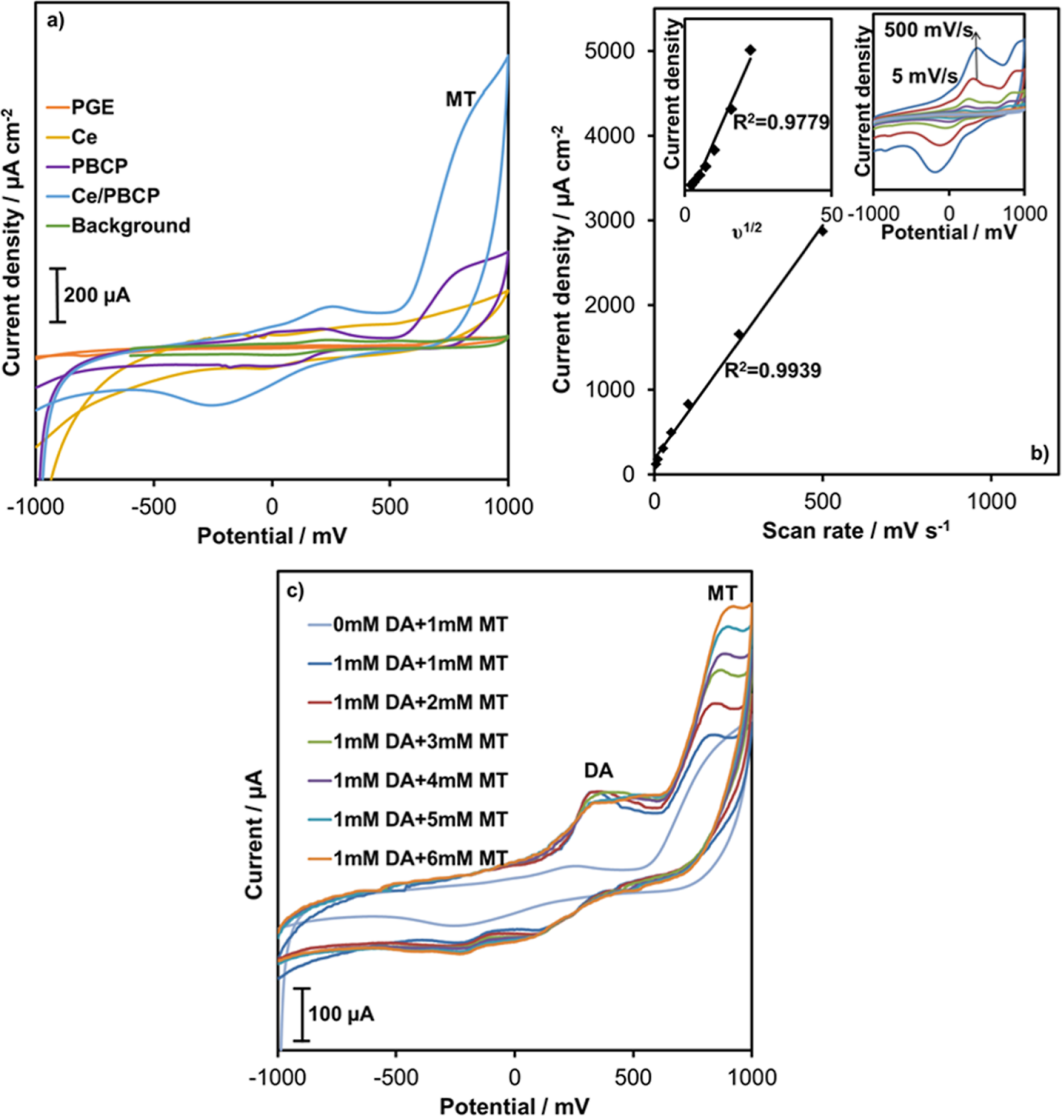
CVs of melatonin oxidation at PGE, Ce, PBCP, and Ce/PBCP electrodes (a). Scan rate vs melatonin oxidation peak current density plot (b) (inset: CVs recorded at different scan rates). CVs of melatonin oxidation in the absence and presence of DA at the Ce/PBCP electrode (c).

Melatonin is an electroactive indolamine species that can be easily oxidized voltammetrically at carbon-based electrodes.^44^ The electrochemical oxidation reaction mechanism of melatonin on the Ce/PBCP electrode is illustrated in Scheme 2. The mechanism likely involves an irreversible oxidation reaction through the indole ring involving the transfer of two electrons and one proton. Furthermore, the dependence of the peak potential (Ep) on pH was investigated to investigate the oxidation mechanism of melatonin. The Ep–pH plot showed a linear change (data not shown). If the slope of the Ep–pH curve were 59 mV/pH, then the oxidation reaction would be associated with reversible electron transfer, involving equal numbers of protons and electrons. However, for our electrode, the slope of the Ep–pH curve was determined to be approximately 35 mV/pH, indicating that the reaction did not involve an equal number of protons and electrons. This electrochemical behavior has also been described and supported in the reports by Levent,^47^ Janegitz,^43^ and Hwa.^48^

**Scheme 2:**
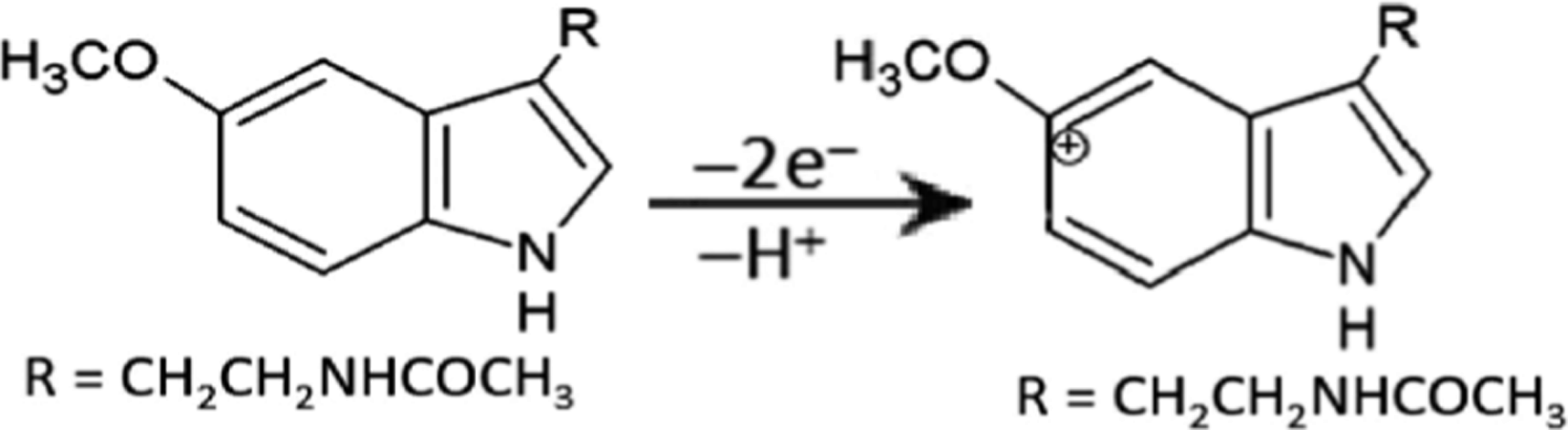
Proposed Mechanism for the Electrochemical Oxidation Reaction of Melatonin

The selective response of the Ce/PBCP electrode to melatonin in the presence of dopamine (DA) as an interfering species was investigated by using CV plots (Figure 4c). For this purpose, the amount of melatonin was changed while keeping the 1 mM DA constant. While the peak current of DA on the Ce/PBCP electrode surface remained constant, the oxidation peak current of melatonin at approximately +820 mV increased depending on the concentration. As shown in Figure 4c, the Ce/PBCP electrode exhibited a highly selective response to melatonin in environments containing interfering species such as DA. This indicates that the produced electrode can be successfully used in samples, such as body fluids.

### Amperometric Detection of Melatonin on the Ce/PBCP Electrode

Sensor performance parameters, such as the limit of detection (LOD) and sensitivity for melatonin detection, were studied using the chronoamperometry technique. For this purpose, melatonin was added to a 10 mL volume of 0.1 M PBS buffer at concentrations ranging from 50 to 2550 μM under 500 rpm constant stirring (Figure 5a). When melatonin was added to the PBS buffer, the current density increased at first and then remained constant. The current also increased in proportion to the increase in the concentration. In addition, a calibration curve was created using the current–time graph (Figure 5b). Thanks to the slope of the calibration curve (S), the sensitivity was calculated as 750.7 μA μM cm^–2^. The sensor’s LOD can be computed by considering the standard deviation (Sy) of the signal in the amperometric curve. Due to the relationship between Sy and S, it is determined by the formula LOD = 3.3­(Sy/S). Accordingly, the LOD value of the sensor was determined as 0.038 μM. The sensor performance exhibited by the Ce/PBCP electrode is compared with that of existing electrodes in the literature, as shown in Table 1. The obtained results showed that the produced sensor is comparable to the currently available electrodes. In addition, a calibration curve was created using the current–time graph (Figure 5b).

**Figure 5:**
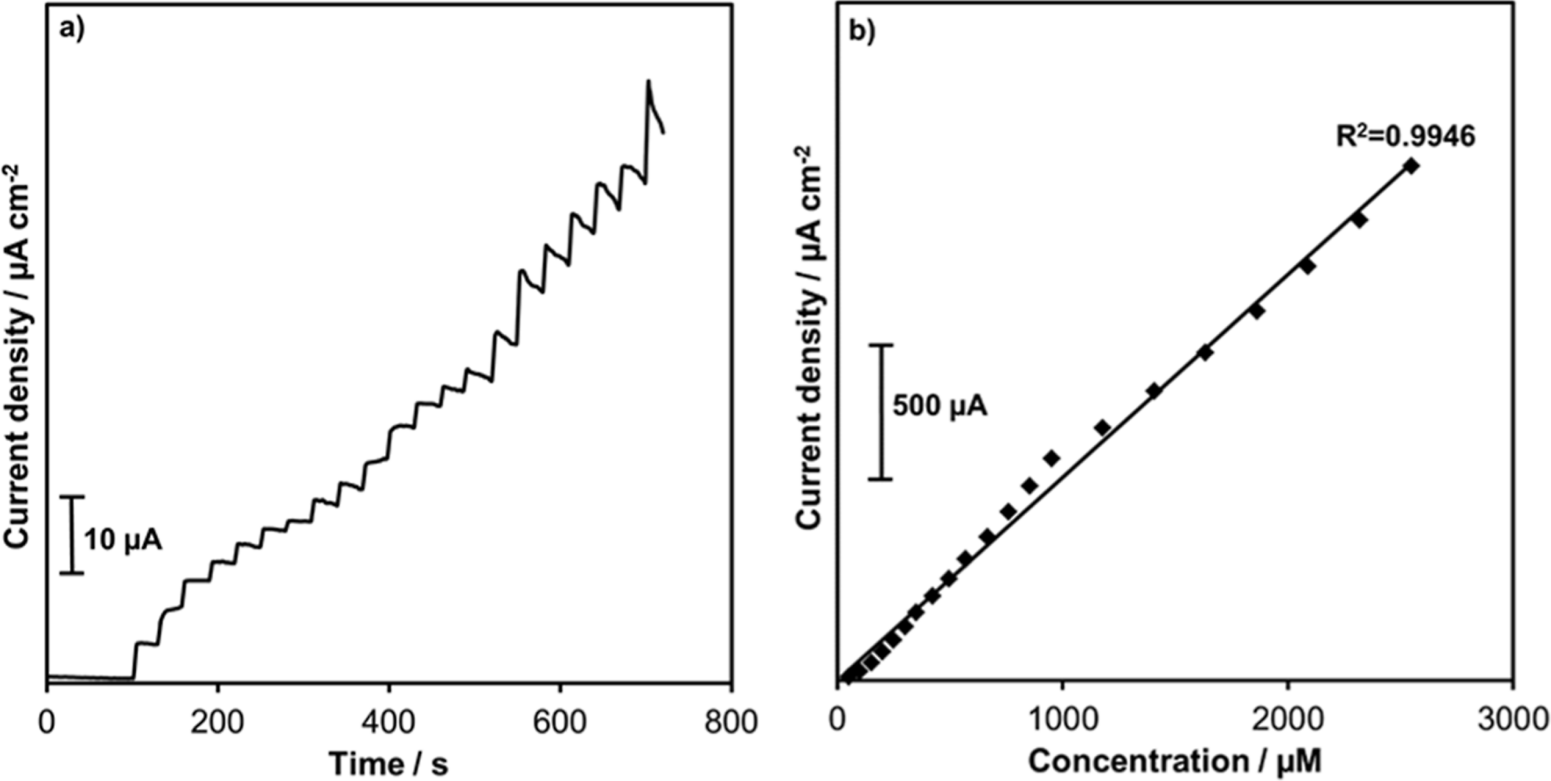
Current density–time response (a) and related calibration curve (b) of the Ce/PBCP electrode for melatonin detection.

**Table 1:** Comparison of Some Sensor Parameters for Melatonin Detection with Different Techniques^a^

electrode	technique	linear range (μM)	LOD (μM)	reference
G-CSPE	amperometry	1–300	0.87	^46^
CNT/SPE	DPV	5–3000	1.1	^49^
PTBO/MWCNTs/GCE	DPV	1–1000	0.027	^50^
BDD	SWV	0.5–4	0.11	^47^
GCE/MnHCF–PEDOT	amperometry	100–4600	100	^51^
Ce/PBCP	amperometry	50–2550	0.038	this study

a G: Graphene; CSPE: carbon screen-printed electrode; CNT: carbon nanotubes; SPE: Screen-printed electrodes; PTBO: polytoluidine blue O; MWCNT: multiwalled carbon nanotube; GCE: glassy carbon electrode; BDD: boron-doped diamond electrode; MnHCF: manganese hexacyanoferrate; PEDOT: poly­(3,4-ethylenedioxythiophene).

The reproducibility of the Ce/PBCP electrode was tested by using seven different electrodes prepared under the same experimental conditions. The melatonin oxidation current response for varying electrode materials is presented in Figure 6b. The recovery % of peak current density was obtained in the range from 93 to 103, indicating that a similar response was observed for varying electrodes.

**Figure 6:**
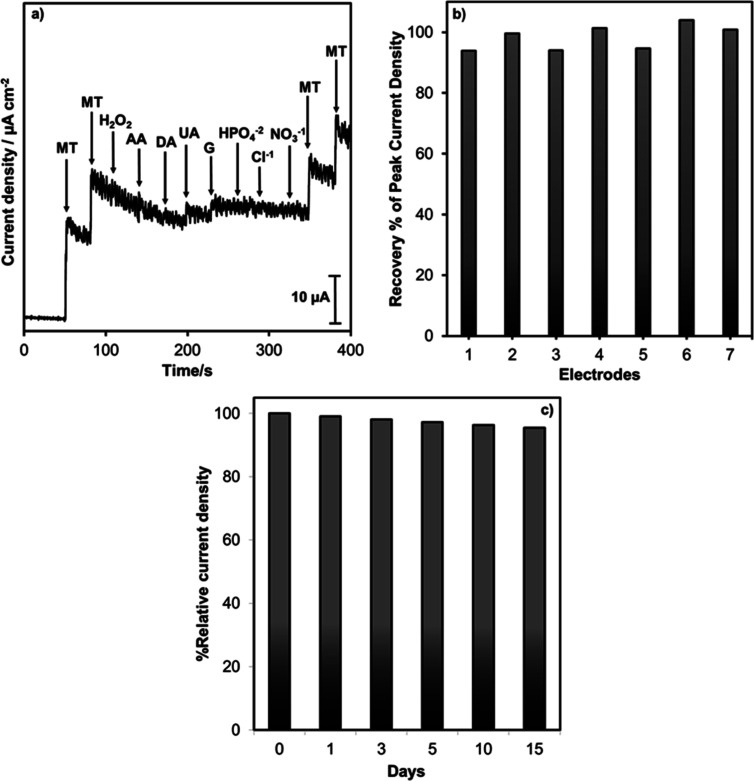
Interference (a), reproducibility (b), and stability (c) test of the Ce/PBCP electrode for melatonin detection.

The stability study was investigated using a consistent oxidation peak current observed in CV plots in a melatonin-containing electrolyte medium for 15 days. The oxidation peak current–day plot generated using the CV curves is depicted in Figure 6c. Despite the 15 day study period, the response of the Ce/PBCP electrode to melatonin remained unchanged. Therefore, the prepared Ce/PBCP electrode exhibited excellent performance in detecting melatonin, with an approximately 4% decrease in current response by the end of the 15th day.

The real sample analysis used turpentine coffee as a sample containing melatonin. The amount of melatonin in turpentine coffee was detected by using the calibration curve obtained amperometrically on the Ce/PBCP electrode surface. For method comparison, the results obtained by the UV–vis technique were compared with those obtained electrochemically (Table 2). UV–vis results and amperometry results were found to be quite consistent with each other, as indicated by the standard deviations obtained from the three analyses. This consistency suggests that the Ce/PBCP electrode can be used for analyzing real food samples.

**Table 2:** Determination of Melatonin Content in the Turpentine Coffee Sample (*n* = 3)

melatonin	concentration (mM)		%recovery	%RSD
	UV–vis amperometry			
	5.83	5.81	99.7	1.4

An interference test was conducted for the effect of possible interfering species originating from coffee (Figure 6a). The ratio of the melatonin concentration to the interfering species concentration was determined as 1:10. The interference test in Figure 6a showed that other interfering species such as hydrogen peroxide (H_2_O_2_), ascorbic acid (AA), dopamine (DA), uric acid (UA), glucose (G), hydrogen phosphate (HPO_4_^2–^), chloride (Cl^–^), and nitrate (NO_3_^–^) did not have a negative effect on melatonin oxidation. The Ce/PBCP electrode exhibited a selective response for melatonin detection in systems containing multiple components such as a coffee sample.

## Conclusion

A PBCP film on a PGE surface was prepared by using electrochemical polymerization and BCP as the monomer. Ce doping was performed on the surface of PBCP to create active surfaces for melatonin detection. XPS, FESEM, EDS, and EIS techniques were used for the characterization of the produced Ce/PBCP electrode. Voltammetric studies showed that Ce doping caused a 3.2-fold increase in melatonin current density compared with PBCP. Moreover, the Ce/PBCP electrode exhibited a highly selective response to melatonin in the presence of an interfering species such as dopamine. Sensor parameters such as LOD and sensitivity were calculated as 0.038 μM and 750.7 μA μM cm^–2^, respectively, using the amperometry technique. The prepared Ce/PBCP electrode exhibited a superior performance for melatonin detection.
